# Effects of DHEA and DHEAS in Neonatal Hypoxic–Ischemic Brain Injury

**DOI:** 10.3390/antiox13121542

**Published:** 2024-12-16

**Authors:** Elena Mayer, Ira Winkler, Eva Huber, Martina Urbanek, Ursula Kiechl-Kohlendorfer, Elke Griesmaier, Anna Posod

**Affiliations:** Department of Pediatrics II (Neonatology), Medical University of Innsbruck, Anichstraße 35, 6020 Innsbruck, Austria

**Keywords:** dehydroepiandrosterone, dehydroepiandrosterone sulfate, mouse model, hypoxia–ischemia, neonatal hypoxic–ischemic brain injury, neuroprotection

## Abstract

Neonatal brain injury remains a significant issue with limited treatment options. This study investigates the potential of the endogenous neurosteroid dehydroepiandrosterone (DHEA) and its sulfate ester (DHEAS) as neuroprotective agents, building on evidence of their mechanisms in adult brain injury models. The primary objective was to evaluate their neuroprotective and anti-oxidative properties in a mouse model of neonatal hypoxic–ischemic brain injury. Using the modified Rice–Vannucci model, brain injury was induced in 7-day-old mouse pups, followed by treatment with various concentrations of DHEA and DHEAS (0.1, 1, and 10 µg/g body weight) via intraperitoneal injection after a 2 h recovery period. Mice were sacrificed after 24 hours for analysis of somatometry, brain injury, apoptosis, microglial activation, and oxidative stress markers (NOX2, 4-HNE, 8-OHdG), along with the anti-oxidant marker SOD1. While no statistically significant effects of DHEA or DHEAS were observed at the tested doses and time points, the absence of toxic or adverse effects highlights their safety profile. These findings provide a foundation for further research into optimizing dosing strategies, timing, and delivery methods. Future studies should refine these variables to maximize neuroprotective efficacy, investigate DHEA(S)’ exact mechanisms of action, and explore their potential for clinical application in neonatal care.

## 1. Introduction

Neonatal encephalopathy is a major cause of mortality and life-long morbidity in newborns [1]. In developed countries, the two most common causes of neonatal brain injury are extremely preterm birth and hypoxic–ischemic encephalopathy (HIE), one of the most severe complications of perinatal asphyxia (PA) [2,3]. PA can be described as a temporary lack of oxygen supply to organ systems following an acute intrapartum hypoxic–ischemic event, which can lead to multi-organ failure in the neonate, potentially affecting the heart, kidneys, lungs, liver, and—often most concerning—the brain [4]. Despite improvements in neonatal care, PA—especially when accompanied by HIE—remains a critical cause of neonatal mortality, accounting for 15% of neonatal deaths worldwide [5]. HIE affects approximately 2 per 1000 live births in developed countries [6,7] with incidence rates up to 15 times higher in low- and middle-income countries [8,9,10]. Among survivors, 25–40% experience long-term neurodevelopmental impairments, such as cerebral palsy, epilepsy, and cognitive deficits [11,12]. These figures highlight the critical need for effective treatments to mitigate HIE’s impact on global neonatal health.

The majority of hypoxic–ischemic events occur intrapartum [8] and tend to affect different regions of the brain depending on gestational age. While the cortex, thalamus, hippocampus, and basal ganglia are mainly harmed in term infants, white matter is primarily injured in preterm infants [13,14]. The neurological damage results from glucose and oxygen deprivation, which causes a primary energy failure and initiates a cascade of biochemical events leading to cell dysfunction, apoptosis, and necrosis of brain cells [13,15]. More specifically, this cascade involves ATP depletion, acidosis, intracellular ion accumulation, neuronal depolarization, uncontrolled release and accumulation of excitatory amino acids, receptor overstimulation leading to massive Ca^2+^ influx and excitotoxicity, mitochondrial dysfunction, production of reactive oxygen species, cell edema, lysis, and death [2,16,17,18,19,20]. The etiopathogenesis of neonatal brain injury is complex and still not fully understood. Current treatments for HIE primarily focus on supportive measures, with therapeutic hypothermia serving as the standard of care to reduce the severity of brain injury. However, no pharmacologic therapies have been established to fully prevent adverse outcomes. This creates a critical research gap, as existing treatments fail to comprehensively address key mechanisms like oxidative stress, neuroinflammation, and excitotoxicity, necessitating the exploration of novel therapeutic approaches.

While therapeutic hypothermia remains the standard of care for HIE, its efficacy is limited in certain populations, such as preterm infants, and its effectiveness decreases in cases of severe HIE. Moreover, its implementation can be challenging in resource-limited settings due to the need for specialized equipment and trained personnel [21,22]. These challenges underscore the urgent need for alternative treatments suitable for a broader range of clinical scenarios.

A specific group of substances acting as sigma-1 receptor (σ1R) agonists has shown great potential for the treatment of neonatal brain injury, as their protective effects have been demonstrated in a variety of brain injury models so far [23,24,25,26,27,28]. One such promising σ1R agonist is the neusteroid hormone dehydroepiandrosterone (DHEA) and its more stable sulfate ester dehydroepiandrosterone sulfate (DHEAS) [29]. In the brain, endogenous DHEA(S) is involved in neurogenesis, neuroprotection, neuronal growth, and survival and has anti-inflammatory, anti-oxidant, anti-glucocorticoid, and anti-apoptotic effects [30,31,32,33,34,35,36,37].

Both DHEA and DHEAS have been studied for their beneficial properties in pathological contexts, such as models of neurodegenerative diseases, adult brain injuries, and ischemia, where they have been shown to protect cells against cell death, neuroinflammation, amyloid beta-protein toxicity, oxidative stress, and excitotoxicity in a timing- and dose-dependent manner [31,32,35,36,37,38,39,40,41,42,43,44,45,46]. However, both substances have also exhibited pro-apoptotic and neurotoxic properties [45,47]. To date, their mechanisms of action are still not fully understood. In the context of hypoxic–ischemic brain injury, several studies have shown that DHEA and DHEAS act primarily through the modulation of a number of receptors, including σ1R, GABA-A receptors, NMDA receptors, NGF receptors, and G-protein-coupled receptors [31,32,34,48,49]. The σ1R regulates cellular Ca^2+^ mobilization from the ER [50] and exerts neuroprotective effects upon activation by inhibiting ischemia-induced glutamate release [51], mitigating Ca^2+^ influx into neuronal cells [52], attenuating neuronal responses to NMDAR stimulation [53], and reducing free radical formation [54]. Additionally, DHEA and DHEA inhibit the expression of several pro-inflammatory cytokines and cytokine-stimulated NF-κB-dependent transcription [35,36,40]. Moreover, DHEA can reduce neuroinflammation and promote anti-inflammatory responses by activating microglial TrkA receptors [30,42]. Given their multi-faceted protective actions, DHEA and DHEAS could potentially fill critical therapeutic gaps by targeting oxidative stress, neuroinflammation, and excitotoxicity in HIE—mechanisms that are only partially addressed by current treatments.

Based on the mechanisms of action and promising previous findings, we aimed to investigate the therapeutic potential of DHEA and DHEAS in a mouse model of neonatal hypoxic–ischemic brain injury, with a particular interest in their anti-oxidative effects. Due to several factors, including similar levels of the hormone in human and rodent brains, the mouse appears to be a suitable model for DHEA(S) studies in central nervous system function and disease [32]. This study is the first to evaluate the in vivo effects of DHEA and DHEAS on neonatal hypoxic–ischemic brain injury, offering new insights into their potential for early therapeutic intervention.

## 2. Materials and Methods

A detailed list of materials and substances used in this study is provided in Supplementary Table S1.

All animal experiments were conducted in strict accordance with current European Union legislation (Directive 2010/63/EU, amending Directive 86/609/EEC) and Austrian law. Prior formal approval for the experiments was obtained from our institution’s Animal Welfare Body and the Austrian Federal Ministry. All efforts were made to minimize the number of animals used and their suffering. CD-1 mice (Charles River Laboratories, Sulzfeld, Germany) were bred and kept at the Central Laboratory Animal Facility, Medical University of Innsbruck, Austria. In total, 209 mouse pups were subjected to hypoxic–ischemic brain injury, which was induced by means of a modified version of the Rice–Vannucci model as described previously [55]. In brief, seven-day-old (P7) CD-1 pups were subjected to right common carotid artery ligation under local anesthesia—by administering lidocaine/prilocaine to the surgical site—and general anesthesia (isoflurane in oxygen, 3.0 vol% for induction). When pups showed a negative toe-pinch reflex, isoflurane was reduced to a maintenance dose of 1.5 vol% in oxygen for the remainder of the procedure. A midline neck incision between the sternocleidomastoid muscle and the trachea was performed. The common carotid artery was looped cranially and caudally with a suture and cut between the double ligatures. After a 90 min recovery period, pups were placed into a controlled chamber and exposed to a hypoxic environment (8% oxygen in nitrogen) for 20 min under normothermic conditions and subsequently returned to their dams. Following a two-hour recovery period, mouse pups were randomly assigned to one of the following treatment groups: (i) control 1× PBS, (ii) solvent control 1× PBS + dimethyl sulfoxide (DMSO), (iii) DHEA 0.1 µg/g, (iv) DHEA 1 µg/g, (v) DHEA 10 µg/g, (vi) DHEAS 0.1 µg/g, (vii) DHEAS 1 µg/g, or (viii) DHEAS 10 µg/g body weight. All mouse pups received a single intraperitoneal (i.p.) injection 2 mm paraumbilically, with an injection volume of 50 μL. They were returned to their dams immediately after treatment until the endpoint assessment.

Sex was determined by visual examination of the anogenital region. Body weight was measured on postnatal day 7 (P7) and postnatal day 8 (P8) using calibrated medical precision scales (Kern/Ascuro Service GmbH, Lörrach, Germany). On P8, mouse pups were sacrificed by decapitation, and brain weight was determined with the same precision scales. Weight gain was calculated as the difference between body weight on P8 and P7, and relative brain weight was determined by dividing brain weight on P8 by body weight on P8. Blood samples were collected in EDTA tubes. Plasma was obtained by centrifugation (1600× g for 15 min at 4 °C), and pooled based on sex to obtain a minimum volume of 100 µL/sample (3–4 animals/sample) and stored at −70 °C until further analysis.

### 2.1. Neuropathological Injury Assessment

For histological endpoint determination and confirmation of successful injury induction, brains were harvested, immersion-fixed in 4% formaldehyde for 72–120 h, paraffin-embedded, and cut into 10 µm thick coronal sections. Neuropathological injury was assessed in Cresyl Violet-stained sections by a blinded observer using a modified scoring system as described previously [56]. Rating was conducted as follows: 0–4 in the cerebral cortex (0, no injury; 1, a few small, isolated groups of injured cells; 2, several larger groups of injured cells; 3, moderate confluent infarction; 4, extensive confluent infarction), and 0–3 for mild, moderate, or severe atrophy and neuronal injury/infarction in the hippocampus, striatum, and thalamus. A total injury score (0–22) was calculated as the sum of all subratings.

### 2.2. Immunohistochemical Analyses

For immunohistochemical endpoints, paraffin-embedded brain sections were deparaffinized and passed through graded alcohols. Endogenous peroxidases were quenched by incubation with 2% H_2_O_2_ in methanol for 30 min. Heat-induced epitope retrieval was performed with a citrate buffer. Unspecific protein blocking was carried out using 5% normal goat serum/1% BSA/0.1% cold fish skin gelatine/0.5% TritonX-100 in 1× TBS/0.05% Tween 20 for 90 min at room temperature. Apoptosis was detected by incubation with rabbit monoclonal anti-cleaved caspase-3 antibody (1:200); activated microglia were detected using rabbit anti-ionized calcium-binding adapter molecule 1 (Iba1, 1:1000). All primary antibodies were diluted in 1%BSA/0.1% cold fish skin gelatine/0.5% TritonX-100 in TBS/0.05% Tween 20 and incubated overnight at 4 °C. After rinsing the slides with PBS, the sections were incubated with biotinylated goat anti-rabbit secondary antibody for 90 min at room temperature, followed by incubation with streptavidin-biotin complex. Enzymatic detection of immunoreactivity was performed using a diaminobenzidine substrate kit for all slides. Staining specificity for all immunohistochemistry protocols was confirmed by including negative controls that underwent identical procedures, except for the omission of primary antibodies. No specific staining was observed in these controls, verifying the specificity of the primary antibodies used.

**Figure 1:**
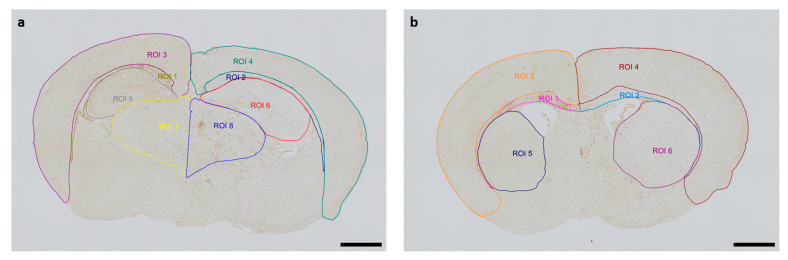
Representative images of regions of interest (ROI) were captured in two section planes. Whole-brain visualization was conducted at 40× magnification (scale bars = 1000 µm). (**a**) Occipital section plane: ROI 1: contralateral white matter, ROI 2: ipsilateral white matter, ROI 3: contralateral cortex, ROI 4: ipsilateral cortex, ROI 5: contralateral hippocampus, ROI 6: ipsilateral hippocampus, ROI 7: contralateral thalamus, ROI 8: ipsilateral thalamus. (**b**) Frontal section plane: ROI 1: contralateral white matter, ROI 2: ipsilateral white matter, ROI 3: contralateral cortex, ROI 4: ipsilateral cortex, ROI 5: contralateral striatum, ROI 6: ipsilateral striatum.

The Allen Mouse Brain Coronal Atlas (available from http://brain-map.org (accessed on 9 October 2024)) was used as an anatomical reference for brain structures. Quantification of cleaved caspase-3-positive cells and activated Iba1-positive microglia in the respective regions of interest (ROI) (cortex and underlying white matter, hippocampus, thalamus, striatum) of both hemispheres was conducted by a blinded observer at two section planes corresponding to occipital coronal level 72 (bregma −1.755 mm) and frontal coronal level 44 (bregma 1.045 mm). Figure 1 shows representative images of all ROIs. The contralateral hemisphere represents the hypoxic-only region, while the ipsilateral hemisphere aligns with the hypoxic–ischemic region. With regard to cleaved caspase-3, cell counts represent absolute numbers of labeled cells. For microglia quantifications, one representative image from each of the two section planes was chosen for each mouse pup. The area of each ROI was manually delineated and measured by an investigator blinded to lesion and treatment. Activated microglia density (D) was calculated by normalization of the count of amoeboid Iba1-positive cells (C) in the ROI to the measured area (A) of the ROI, also considering the thickness (T) of the section: [D = C/(AxT)]. The analyses were performed using an Olympus IX83 microscope equipped with a DP27 color camera (Olympus Austria GmbH, Vienna, Austria).

### 2.3. Protein Fractionation and Western Blotting

For protein endpoint studies, brains were harvested, snap-frozen in liquid nitrogen, and stored at −80 °C until further use. Isolation of cytosolic, nuclear, and mitochondrial protein fractions was performed according to a modified protocol by Dimauro et al. [57], based on protocols from Cox and Emili [58]. Briefly, brains were homogenized in an STM buffer (250 mM sucrose, 50 mM Tris–HCl pH 7.4, 5 mM MgCl_2_) with protease inhibitors (1× complete EDTA-free protease inhibitor cocktail and 1 mM phenylmethylsulfonyl fluoride (PMSF)) and centrifuged at 800× g for 15 min at 4 °C producing a supernatant containing cytosolic and mitochondrial fractions as well as a pellet containing a nuclear fraction. The nuclear pellet was centrifuged two more times in the STM buffer following resuspension in the NET buffer (20 mM HEPES pH 7.9, 1.5 mM MgCl_2_, 500 mM NaCl, 0.2 mM EDTA pH 8.0, 10% glycerol, 1% Triton X-100 with protease inhibitors) and kept on ice for 30 min. The supernatant with cytosolic and mitochondrial fractions was centrifuged two more times in the STM buffer at 11,000× g for 10 min at 4 °C. The resulting supernatant containing only cytosolic fractions was transferred to a new tube while the mitochondrial pellet was resuspended in the ME buffer (20 mM Tris-HCl pH 7.4, 400 mM NaCl, 10% glycerol, 1% Triton X-100 with protease inhibitors) and kept on ice for 30 min. The nuclear and mitochondrial fractions were sonicated three times on ice at a high setting for 4–10 s and subsequently centrifuged at 9000× g for 30 min at 4 °C. Supernatants containing nuclear or mitochondrial fractions were then transferred to their respective new tubes. Protein quantification of all three fractions was performed using the BCA Protein Assay Kit (Pierce/Fisher Scientific GmbH, Vienna, Austria). Fractionated samples were denatured by heating at 95 °C for 5 min. A total of 15–20 µg of proteins was separated by SDS-polyacrylamide gel electrophoresis using stain-free gels and transferred to PVDF membranes. Membranes were blocked for 1–2 h in 5% non-fat milk and incubated with primary antibodies against superoxide dismutase 1 (SOD1) or NADPH oxidase 2 (NOX2) overnight at 4 °C. After washing, membranes were incubated with specific horseradish peroxidase-conjugated secondary antibodies, and bands were developed with an ECL solution. Signal density quantification was performed using Bio-Rad ImageLab Software 6.1. To account for loading discrepancies or variations in protein transfer, signal densities for SOD1 and NOX2 were normalized using β-Actin as a loading control. 

### 2.4. ELISA of 4-Hydroxynonenal and 8-Hydroxydesoxyguanosin

Plasma concentrations of 4-Hydroxynonenal (4-HNE) and 8-Hydroxydesoxyguanosin (8-OHdG) were determined by using competitive enzyme-linked immunosorbent assays (ELISAs) according to the manufacturer‘s instructions. Briefly, pooled mouse plasma was plated in duplicates on 96-well plates coated with 4-HNE or 8-OHdG, respectively. Samples were added in a 1:20 dilution for 8-OHdG and undiluted for 4-HNE. Simultaneously, a biotinylated detection antibody specific for 4-HNE and a horseradish peroxidase (HRP)-conjugated detection antibody for 8-OHdG were added to the wells. After incubation, plates were washed several times with a washing buffer. For 4-HNE, an HRP-conjugated antibody was added to the plate and again washed after further incubation. Afterwards, tetramethylbenzidine (TMB) was pipetted into the wells, and an enzymatic color reaction was developed for 15 to 20 min in the dark. The chromogenic reaction was stopped using sulfuric acid, and color development was measured at 450 nm using a Hidex Sense Microplate Reader (HVD Life Sciences, Vienna, Austria). Sample concentration was determined in ng/mL by employing a calibration curve. The calibration curve for 4-HNE and 8-OHdG ranged from 0.63 to 40 ng/mL and from 0.94 to 60 ng/mL, respectively.

### 2.5. Statistical Analysis

Statistical analysis was performed using SPSS version 29.0 for Windows (IBM Corporation, Armonk, New York, NY, USA) and GraphPad Prism version 10.1.2 for Windows (GraphPad Software, Boston, MA, USA). Data distribution was evaluated by means of histogram analysis as well as the Shapiro–Wilk test. If data were normally distributed and no more than two groups were compared at a time, Student’s *t* test was applied. The equality of variances was assessed with Levene’s test. Heterogeneity of variances prompted utilization of a modified *t* test. For analysis of data not belonging to a particular distribution, a Mann–Whitney U test was applied. If more than two groups were compared at a time, overall differences between groups were detected with an analysis of variance (ANOVA) in the case of normal distribution or the Kruskal–Wallis test for non-normally distributed data. Post-hoc analysis was conducted by means of Tukey’s or Mann–Whitney U test with Bonferroni correction for multiple comparisons. Results were regarded as statistically significant when *p* < 0.05.

## 3. Results

### 3.1. Study Population

**Figure 2:**
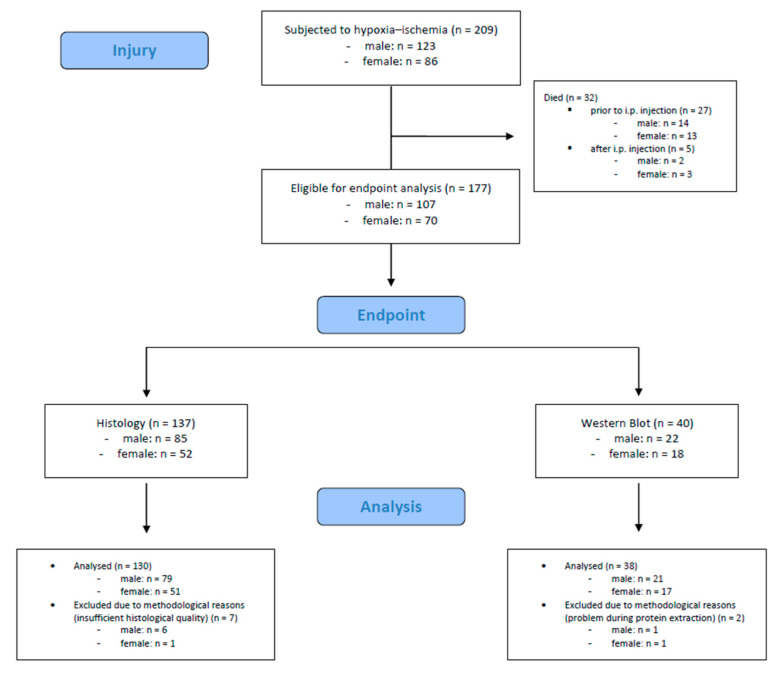
Flow diagram illustrating the experimental workflow and animal cohort distribution.

A total number of 209 mouse pups were subjected to hypoxic–ischemic injury. Of these, 32 (15.3%) died during or shortly after the procedure (*n* = 27 died prior to i.p. injection, *n* = 5 died after i.p. injection). The remaining 177 mouse pups were eligible for endpoint assessments. We included 168 mouse pups (male: *n* = 100, female: *n* = 68) in the final analysis; 9 mouse pups were excluded due to methodological reasons. Detailed information is provided in Figure 2.

#### Somatometry

Somatometric data were obtained from all animals included in the endpoint analysis (n = 168) as a general marker of overall health and metabolic stress. Mean ± standard deviation (SD) body weight on P7 was 4.9 ± 0.5 g; mean ± SD body weight on P8 was 5.3 ± 0.8 g; median (25th; 75th percentile) weight gain was 0.5 (0.2; 0.7) g; median (25th; 75th percentile) brain weight was 0.21 (0.20; 0.22) g; and median (25th; 75th percentile) relative brain weight was 0.040 (0.037; 0.043). Females were lighter on P7 (*p* = 0.052) and P8 (*p* = 0.045), but weight gain and (relative) brain weight did not differ between sexes (all *p* > 0.05). No overall significant differences were detected in any somatometric parameters with regard to treatment (all *p* > 0.05).

### 3.2. Neuropathological Injury

**Figure 3:**
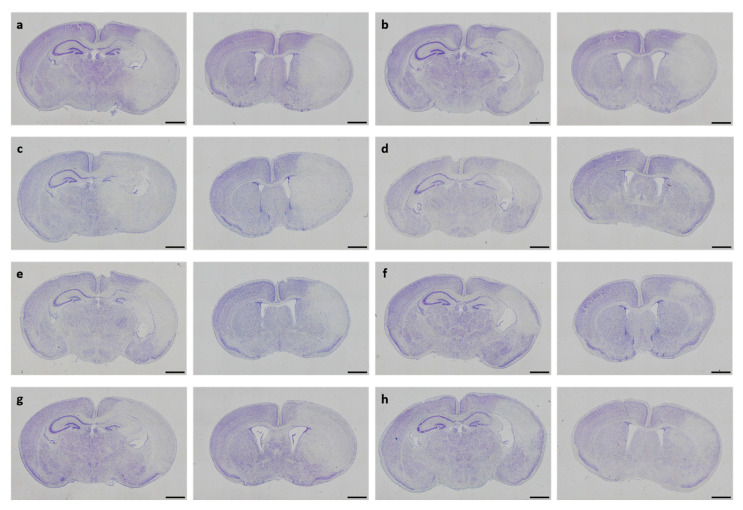
Neuropathological injury assessment. Representative images of Cresyl Violet-stained coronal brain sections displaying hypoxic–ischemic injury in different treatment groups (**a**–**h**). Whole-brain visualization was performed using a 40-fold magnification (scale bars = 1000 µm). Two coronal section planes corresponding to coronal level 72 (bregma −1.755 mm, left) and coronal level 44 (bregma 1.045 mm, right) are displayed per brain. Treatment groups are as follows: (**a**) control 1× PBS, (**b**) solvent control 1× PBS + DMSO, (**c**) DHEA 0.1 µg/g bodyweight (bw), (**d**) DHEA 1 µg/g bw, (**e**) DHEA 10 µg/g bw, (**f**) DHEAS 0.1 µg/g bw, (**g**) DHEAS 1 µg/g bw, (**h**) DHEAS 10 µg/g bw.

In neonatal CD-1 mice subjected to hypoxia–ischemia and treated i.p. after insult, a blinded observer assessed neuropathological injury using an established scoring system. No overall statistical differences in neuropathological injury extent were detected in the subscores or total injury score (all *p* > 0.05). Details regarding total injury scores are provided in Table 1. Representative images of Cresyl Violet stainings for neuropathological injury assessment are shown in Figure 3. No sex-specific differences were observable (all *p* > 0.05).

### 3.3. Anti-Apoptotic Potential of DHEA and DHEAS

**Figure 4:**
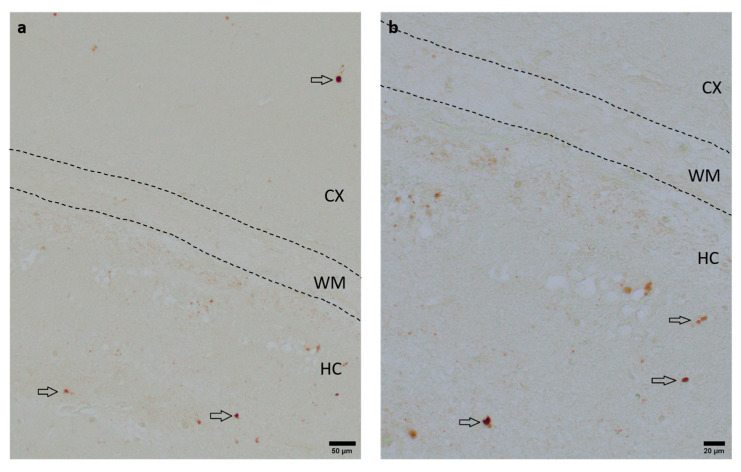
Representative photomicrographs of activated caspase-3-positive cells in the occipital section plane cortex (CX), white matter (WM), and hippocampus (HC) (coronal level 72, bregma −1.755 mm). Arrows indicate positive cells. Visualization was performed using a 100-fold (scale bar = 50 µm) (**a**) and 200-fold magnification (scale bar = 20 μm) (**b**).

To assess the neuroprotective potential of DHEA and DHEAS against apoptosis, the immunohistochemical quantification of activated caspase-3-positive cells was performed in six mouse pups per treatment group. Detection of activated caspase-3 is considered a reliable marker for apoptotic or pre-apoptotic cells [59]. Analysis was conducted on two coronal section planes, examining both the contralateral (hypoxic-only) hemisphere and the ipsilateral (hypoxic–ischemic) hemisphere. Representative photomicrographs of activated caspase-3 immunohistochemistry are shown in Figure 4. No significant overall differences in caspase-3 activation were detectable. Details are provided in Table 2. No sex-specific differences in caspase-3 activation were observed in any brain region across all treatment groups (all *p* > 0.05).

### 3.4. Microglial Cell Activation

**Figure 5:**
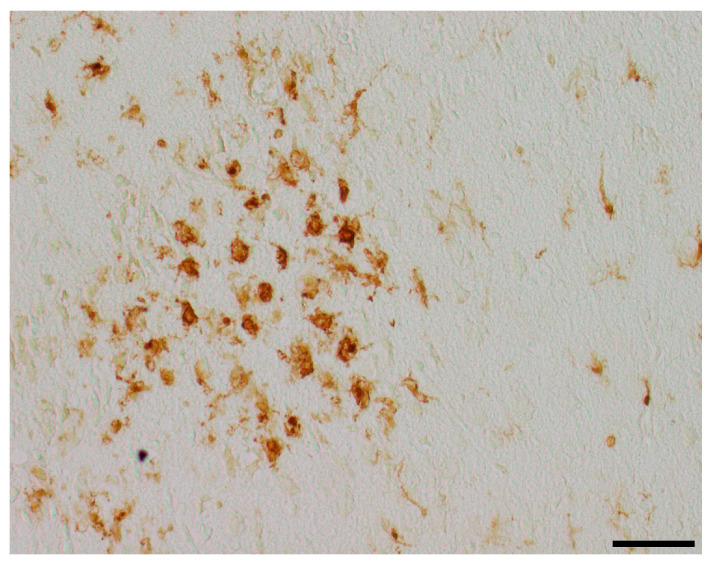
Representative photomicrographs of activated Iba1-positive microglial cells in the occipital section plane cortex (coronal level 72, bregma −1.755 mm). Visualization was performed using a 200-fold (scale bar = 50 µm) magnification.

**Figure 6:**
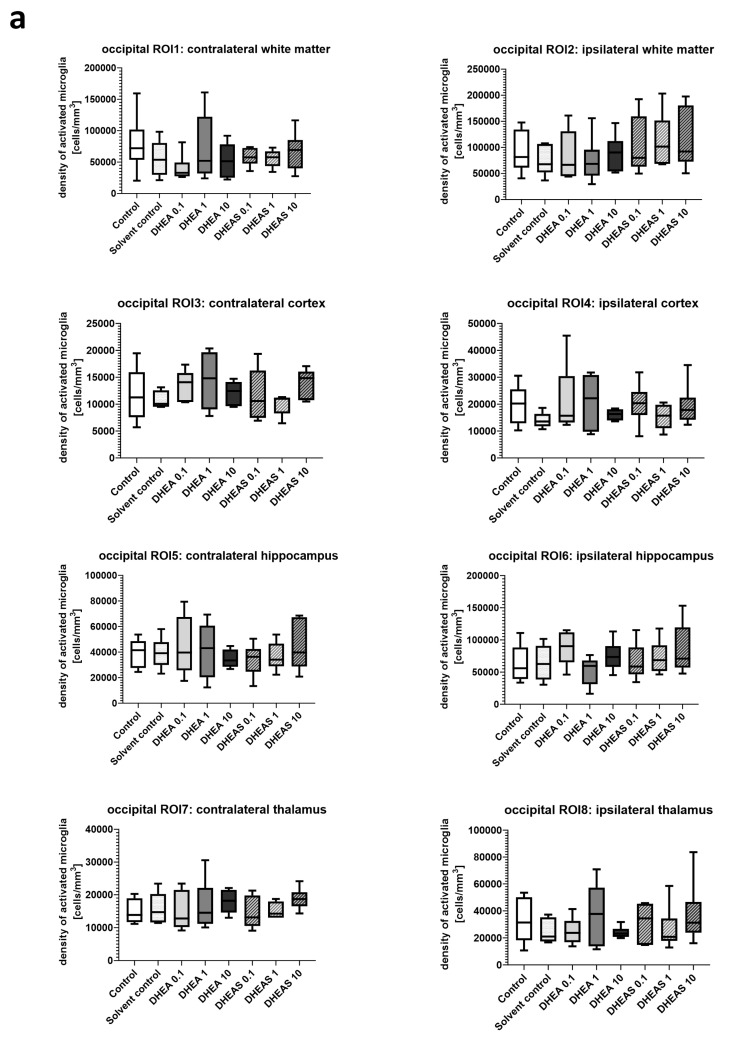

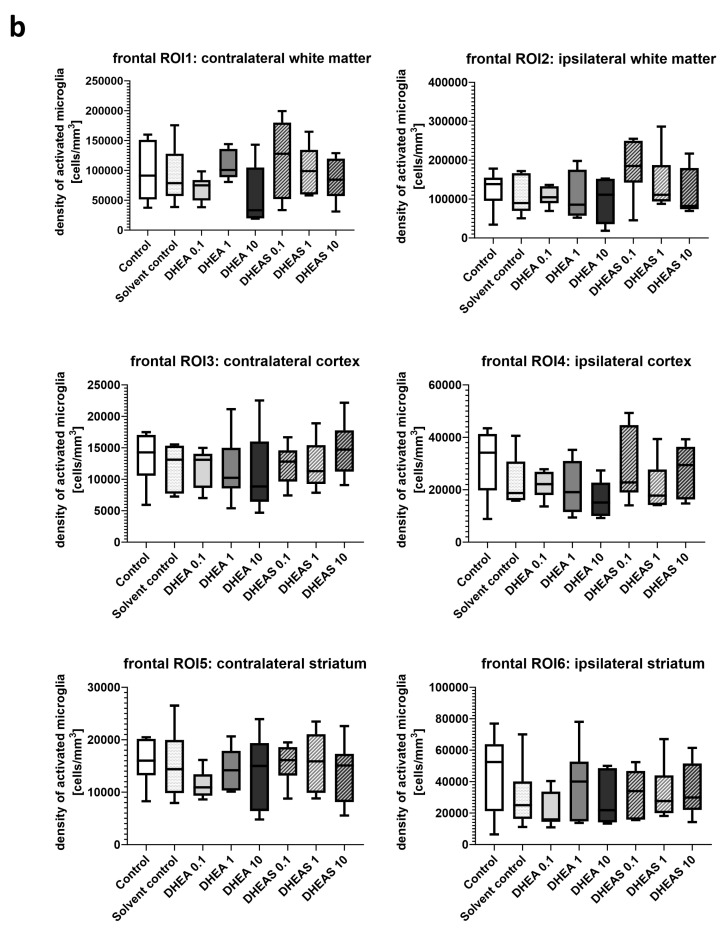
Microglial activation in different brain regions in the occipital (**a**) and frontal (**b**) section plane. Occipital section plane (**a**) corresponds to coronal level 72 (bregma −1.755 mm). Frontal section plane (**b**) corresponds to coronal level 44 (bregma 1.045 mm). Numbers in the different treatment groups indicate concentrations in µg/g bodyweight. Center lines in the boxes represent medians, box edges mark 1st and 3rd quartiles, and whiskers indicate 10th and 90th percentiles. No overall significant differences in microglial cell activation in the analyzed brain regions were detected (Kruskal–Wallis test, all *p* > 0.05). Animals per group: n = 6.

Inflammation has been shown to play a critical role in neonatal HIE. As the innate immune cells of the CNS, microglia patrol the brain parenchyma to ensure brain homeostasis, defend the brain against pathogens, and are important mediators of neuroinflammation. They undergo a dynamic process of morphological changes to respond to different stimuli, such as hypoxia–ischemia, and are therefore an important cell type for investigation [60,61]. To assess microglial cell activation, the immunohistochemical quantification of activated Iba1-positive microglia was performed in six mouse pups per treatment group. The activation status of each cell was determined based on its amoeboid morphology. The density of Iba1-positive cells was calculated for each ROI to account for differences in the size of brain regions. Analysis was conducted on two coronal section planes, examining both the contralateral (hypoxic-only) hemisphere and the ipsilateral (hypoxic–ischemic) hemisphere. Representative photomicrographs of activated Iba1-positive microglial cells are shown in Figure 5. Although brain regions exposed to hypoxia–ischemia generally showed stronger glial activation compared to hypoxic-only regions, microglial activation did not differ significantly between treatment groups (all *p* > 0.05) (Figure 6). No sex-specific differences in activated microglia density were observed in any brain region across all treatment groups (all *p* > 0.05).

### 3.5. Anti-Oxidative Potential of DHEA and DHEAS

#### 3.5.1. Upstream Markers of Oxidative Stress—Western Blot

**Figure 7:**
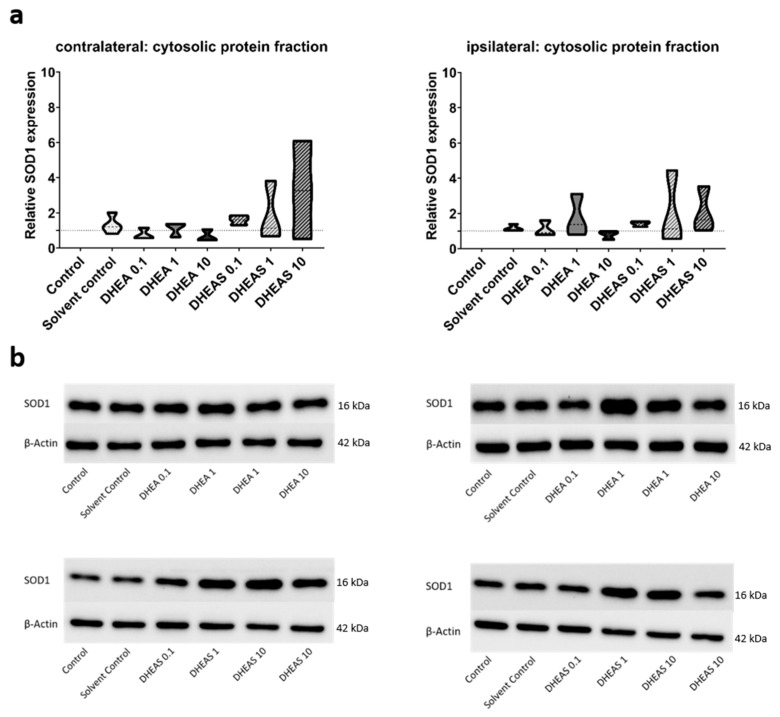
Relative SOD1 expression across treatment groups. Numbers in the different treatment groups indicate concentrations in µg/g bodyweight. (**a**) Truncated violin plots depict the distribution of relative SOD1 expression levels in different treatment groups in the cytosolic protein fractions of the contralateral and the ipsilateral hemispheres, with the control group set to 1 (dotted line). The width of the violins corresponds to the frequency of observations at each expression level. The extreme tails of the distributions were truncated to highlight the central portion of the data, emphasizing the median (dashed lines), the interquartile range (black lines), and the overall shape of the distribution. The control group serves as the baseline for comparison, and its distribution is centered around 1. Number of pooled samples per group: n = 3–4. (**b**) Exemplary Western blots showing SOD1 reactivity in different treatment groups in the contralateral (left) and ipsilateral hemisphere (right). β-Actin served as a loading control.

**Figure 8:**
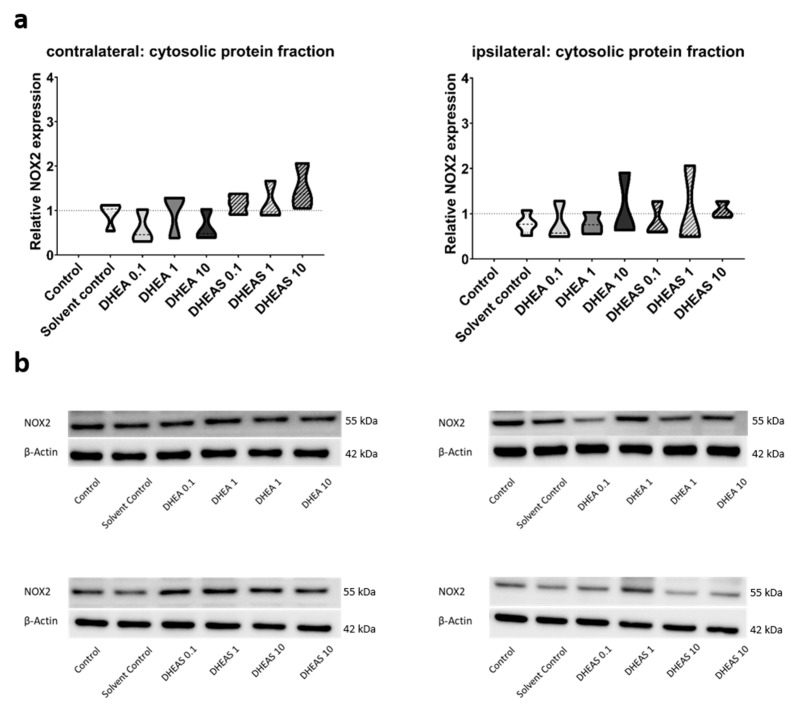
Relative NOX2 expression across treatment groups. Numbers in the different treatment groups indicate concentrations in µg/g bodyweight. (**a**) Truncated violin plots depict the distribution of relative NOX2 expression levels in different treatment groups in cytosolic protein fractions of the contralateral and the ipsilateral hemispheres, with the control group set to 1 (dotted line). The width of the violins corresponds to the frequency of observations at each expression level. The extreme tails of the distributions were truncated to highlight the central portion of the data, emphasizing the median (dashed lines), the interquartile range (black lines), and the overall shape of the distribution. The control group serves as the baseline for comparison, and its distribution is centered around 1. Number of pooled samples per group: n = 3–4. (**b**) Exemplary Western blots showing NOX2 reactivity in different treatment groups in the contralateral (left) and ipsilateral hemispheres (right). β-Actin served as a loading control.

To evaluate the anti-oxidative potential of DHEA and DHEAS, we analyzed two upstream markers of oxidative stress, SOD1 and NOX2, in pooled protein extracts of each brain hemisphere via Western blot. SOD1 constitutes a front-line defense mechanism against oxidative stress, acting as an anti-oxidant, while NOX2 is a major source of oxidative stress [62,63]. Since SOD1 and NOX2 are expected to be primarily present in cytosolic protein fractions, only the results of this fraction are presented. Although a trend towards increased SOD1 expression could be observed in DHEA(S)-treated animals, no statistically significant differences in SOD1 expression were detected in any protein fraction of either hemisphere across all treatment groups (all *p* > 0.05) (Figure 7). Similarly, no significant effects of the treatments on NOX2 expression were observed (all *p* > 0.05) (Figure 8). Sex-specific differences were not assessed due to limited case numbers.

#### 3.5.2. Downstream Markers of Oxidative Stress—ELISA

**Figure 9:**
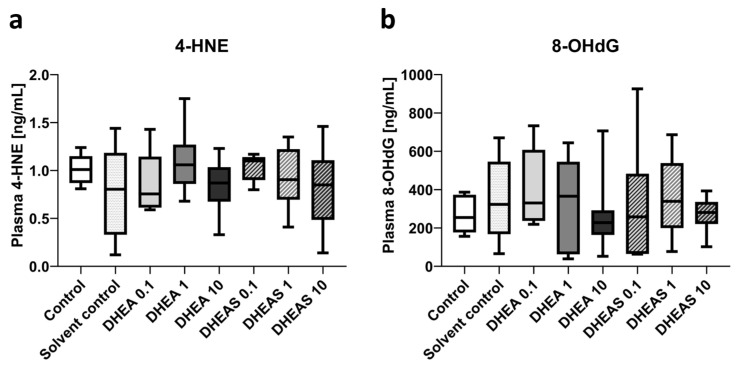
Plasma concentrations of oxidative stress markers 4-HNE (**a**) and 8-OHdG (**b**). Numbers in the different treatment groups indicate concentrations in µg/g bodyweight. Center lines in the boxes represent medians, box edges mark 1st and 3rd quartiles, and whiskers indicate 10th and 90th percentiles. (**a**) No overall significant differences in 4-HNE plasma concentrations were detected. Number of pooled samples per group: n = 5–6. (**b**) No overall significant differences in 8-OHdG plasma levels were detected. Number of pooled samples per group: n = 6–8.

To assess oxidative stress also downstream, we quantified 4-HNE and 8-OHdG in pooled plasma samples using ELISAs. The compound 4-HNE is considered one of the major mediators of oxidative stress and an important downstream marker of lipid peroxidation [64]. The nucleotide 8-OHdG is one of the major DNA damage types induced by oxidative stress and therefore considered a pivotal marker of oxidative DNA damage [65]. Plasma concentrations of both tested oxidation markers showed no overall significant differences across treatment groups (all *p* > 0.05) (Figure 9). In addition, no sex-specific differences were detectable (all *p* > 0.05).

## 4. Discussion

Hypoxic–ischemic brain injury continues to be a major cause of mortality and morbidity among neonates worldwide. Despite this, little progress has been made in pharmacologic treatment options over the past few decades, with most being limited to supportive measures. This highlights the importance of finding additional therapeutic agents for treating neonatal HIE.

In previous research, both DHEA and DHEAS have shown neuroprotective, anti-apoptotic, and anti-inflammatory properties in adult models of brain injury, overall demonstrating great therapeutic potential [38,39,42,43,44,46,49]. DHEA(S) has also shown promise in other human pathologies such as glioma [66], rheumatic disease [67], and postmenopausal symptoms [68]. This study aimed to evaluate the neuroprotective efficacy of DHEA and DHEAS, specifically focusing on their anti-oxidative effects in a neonatal model of hypoxic–ischemic brain injury.

In this study, the neuroprotective and anti-oxidative properties of DHEA and DHEAS were explored at various doses (0.1 µg, 1 µg, or 10 µg per g body weight), with no significant reduction in injury scores, apoptosis, microglial activation, or oxidative stress markers across the groups. However, we demonstrated that DHEA and DHEAS have no negative impact on injured brains. The absence of observable therapeutic benefits in reducing oxidative stress and neuronal damage might stem from a number of factors, including the dosing strategy. While neurosteroids like DHEA and DHEAS often exhibit dose-dependent effects and the beneficial effects of both substances have been shown to be highly timing- and dosage-dependent, there is no consensus regarding the optimal conditions for neurosteroid use in similar models [32,38,39,41,43,44,45,46]. Previous studies suggest an inverted U-shaped dose–response curve for DHEA and DHEAS, where moderate concentrations yield the best results, while excessively high or low doses can be either ineffective or even neurotoxic [32]. Importantly, much of this research has been conducted in adult rodents, where doses ranging from 20 to 100 mg/kg were found to be effective [41,44,45,69,70]. This study used lower doses due to the solubility limitations of DHEA(S) in PBS and concerns regarding the potentially harmful effects of the solvent DMSO [71]. Consequently, the administered doses may not have achieved the concentrations necessary for eliciting a neuroprotective anti-oxidative response.

Here, DHEA and DHEAS were administered via intraperitoneal injection after a two-hour recovery period post-injury. While intraperitoneal administration is practical for neonatal models, it also comes with its limitations. Beyond intraperitoneal injection [45], other studies employed oral [41], intravenous [46], and subcutaneous [70,72] administration, as well as subcutaneous pellets [44,69], showing evidence of neuroprotection. Exploring alternative administration routes could improve DHEA(S) bioavailability and enhance blood–brain barrier penetration. A more secure application method may also prevent leakage at the injection site. Moreover, repeated or continuous dosing could sustain therapeutic levels longer, potentially amplifying neuroprotective effects.

Therefore, higher dosages or a different route of administration may enhance the permeability and effectiveness of DHEA(S). 

Additionally, the timing of treatment plays a pivotal role in neuroprotective efficacy. In this study, the compounds were administered during the latent phase of HIE, which is considered optimal for interventions aimed at preventing secondary injury. However, there is no consensus regarding optimal timing of DHEA(S) treatment in the literature, with some studies demonstrating significant protective effects when either administered before, during or within minutes after injury, while others employed continuous or repeated dosing strategies post-injury [39,44,46,69,70,72,73,74]. Contrary to this, Li and colleagues reported neurotoxic effects when administered too early and showed that treatment 3 to 48 h after injury yielded the best outcome [45]. This lack of consensus highlights the need for future studies investigating whether early, delayed, or repeated administration of DHEA(S) can improve brain damage in neonates.

Another notable limitation in the present study is the early endpoint analysis conducted 24 h after the hypoxic–ischemic event. While this timeframe captures early injury markers, it may not reveal the full extent of DHEA(S)’s potential.

Neuroprotective agents often require extended periods to induce complex biological changes that promote cell survival, tissue repair, and functional recovery, particularly in the brain, where injury responses unfold in distinct temporal stages. Longer observation periods enable assessment of secondary and tertiary injury phases, including delayed cell death, resolution of neuroinflammation, and regenerative processes occurring over days to weeks. DHEA(S) may progressively mitigate oxidative stress and inflammation, with cognitive and behavioral benefits potentially emerging as structural reorganization of the brain occurs. Since other studies involving longer observation periods (up to several weeks) have shown that delayed administration of DHEA(S) can lead to improvements in cognitive and behavioral outcomes, we cannot rule out that our limited 24 h window may have overlooked subtle anti-oxidative and neuroprotective benefits that could become apparent in a more extended timeframe [41,44,45,69,70,72].

One major factor likely contributing to the inconclusive results is the inherent variability in the experimental model used in this study. The modified Rice–Vannucci model is known for a certain degree of variability in resulting brain injuries, and despite efforts to standardize conditions, variations in injury severity and treatment responses were still observed among the individual pups, potentially confounding the outcomes. This variability is consistent with the challenges of studying neuroprotective agents in neonatal models, where factors such as the sex of the pups, differences in brain development, and individual susceptibility to oxidative stress play significant roles, further complicating detection of distinct effects.

Moreover, the study’s small sample size may have limited the statistical power to detect subtle anti-oxidative effects, particularly given the inherent variability within the model. More samples in each analyzed group could have possibly revealed more nuanced effects. This, for example, is indicated by the results of SOD1. A trend towards increased SOD1 expression could be observed in DHEA(S)-treated animals, but—likely due to the small sample size of n = 3 for these groups—the results were not significant. While this trend suggests a potential anti-oxidative response to DHEA(S) treatment, the lack of statistical significance prevents definitive conclusions. In some instances, samples from mice receiving the same treatment were also pooled due to limited protein extraction quantities, potentially diluting individual differences and masking the anti-oxidative effects of DHEA and DHEAS. Additionally, 4-HNE concentrations in plasma sample pools were near the detection limit, complicating result interpretation.

Finally, the unexpected absence of neuroprotective effects in this study may also be attributed to the differences between the maturing brain of a newborn and the adult brain. In recent years, research has shown that the neonatal brain is not just a smaller version of the adult brain, and therefore the pathophysiology as well as the therapeutic approach to its injury may differ greatly [75].

Future studies should focus on elucidating the protective role of DHEA and DHEAS through targeted experimental approaches. For instance, further in vitro studies could investigate their effects on oxidative stress, neuroinflammation, and excitotoxicity in primary neuronal or glial cell cultures. Additionally, based on the findings of Hübner and colleagues, who observed neuroprotective effects of DHEA in an oligodendrocyte cell line [76], future in vivo studies should explore the impact of DHEA on white matter integrity. Given that oligodendrocytes are key cell types in white matter [77], such investigations may reveal critical insights into DHEA’s therapeutic potential for preserving or restoring white matter function, particularly in neonatal hypoxic–ischemic brain injury.

## 5. Conclusions

In conclusion, this study did not observe significant anti-oxidative neuroprotective effects of DHEA or DHEAS at the tested doses and timing in a neonatal hypoxic–ischemic brain injury model. However, several factors, including the route of administration, dosing strategies, timing of intervention, and early endpoint analysis, may have contributed to the lack of observable effects. Future research should not only optimize these parameters but also investigate the specific mechanisms by which DHEA(S) exerts neuroprotection, such as targeting oxidative stress, neuroinflammation, and excitotoxicity.

Given their well-documented anti-oxidative properties in adult models, these neurosteroids remain promising candidates for neonatal neuroprotection. Further studies should explore higher or repeated doses, alternative administration routes, and extended observation periods to fully capture the long-term benefits of DHEA(S). In particular, future research should examine DHEA’s effects on white matter integrity, with a focus on its impact on oligodendrocytes, a critical cell type in white matter that is highly vulnerable to neonatal brain injury. Additionally, future investigations should account for sex-specific responses and aim to identify biomarkers that can predict individual treatment efficacy in neonatal populations. Refining DHEA and DHEAS therapies could have significant clinical implications, particularly for early intervention in neonatal brain injury, where timely neuroprotection may critically influence developmental outcomes.

Although this study did not conclusively demonstrate the anti-oxidative neuroprotective potential of DHEA or DHEAS, it found no detrimental effects. It also highlights critical areas for refinement in future neuroprotection studies targeting neonatal hypoxic–ischemic brain injury.

## Figures and Tables

**Table 1:** Neuropathological injury assessed in Cresyl Violet-stained sections.

Treatment Group	Number of Animals, n	Total Injury Score, Median (25th; 75th Percentile)	*p*-Value
Control	16	3.5 (1.0; 10.5)	
Solvent control	16	8.3 (0.9; 12.0)	
DHEA 0.1 µg/g bw	15	5.0 (0.5; 13.0)	
DHEA 1 µg/g bw	17	9.5 (1.5; 12.0)	
DHEA 10 µg/g bw	16	5.0 (1.1; 7.9)	
DHEAS 0.1 µg/g bw	16	7.5 (1.3; 12.8)	
DHEAS 1 µg/g bw	17	4.0 (1.3; 11.0)	
DHEAS 10 µg/g bw	17	6.5 (1.5; 12.5)	0.829 ^1^

*Abbreviations: DHEA*, dehydroepiandrosterone; *DHEAS*, dehydroepiandrosterone sulfate; *bw*, body weight. ^1^ Overall statistical differences assessed by Kruskal–Wallis test (H_(7)_ = 3.561).

**Table 2:** Number of activated caspase-3-positive cells per brain region.

Section Plane	Hemisphere	Brain Region	Treatment Group	Number of Positive Cells, Median (25th; 75th Percentile)	*p*-Value
Occipital	Contralateral	White matter			
			Control	11 (4; 12)	
			Solvent control	6 (4; 10)	
			DHEA 0.1 µg/g bw	6 (4; 11)	
			DHEA 1 µg/g bw	13 (11; 16)	
			DHEA 10 µg/g bw	10 (6; 14)	
			DHEAS 0.1 µg/g bw	11 (3; 23)	
			DHEAS 1 µg/g bw	7 (6; 10)	
			DHEAS 10 µg/g bw	8 (6; 11)	0.268 ^1^
	Ipsilateral	White matter			
			Control	125 (12; 181)	
			Solvent control	16 (4; 162)	
			DHEA 0.1 µg/g bw	19 (11; 111)	
			DHEA 1 µg/g bw	182 (10; 282)	
			DHEA 10 µg/g bw	27 (10; 141)	
			DHEAS 0.1 µg/g bw	121 (7; 248)	
			DHEAS 1 µg/g bw	25 (9; 228)	
			DHEAS 10 µg/g bw	83 (17; 230)	0.836 ^1^
	Contralateral	Cortex			
			Control	16 (13; 25)	
			Solvent control	16 (9; 20)	
			DHEA 0.1 µg/g bw	23 (14; 33)	
			DHEA 1 µg/g bw	24 (13; 32)	
			DHEA 10 µg/g bw	15 (9; 24)	
			DHEAS 0.1 µg/g bw	20 (13; 24)	
			DHEAS 1 µg/g bw	20 (16; 26)	
			DHEAS 10 µg/g bw	19 (15; 23)	0.590 ^1^
	Ipsilateral	Cortex			
			Control	160 (37; 276)	
			Solvent control	37 (10; 202)	
			DHEA 0.1 µg/g bw	39 (25; 107)	
			DHEA 1 µg/g bw	286 (23; 376)	
			DHEA 10 µg/g bw	34 (24; 220)	
			DHEAS 0.1 µg/g bw	158 (18; 446)	
			DHEAS 1 µg/g bw	39 (27; 169)	
			DHEAS 10 µg/g bw	93 (25; 366)	0.901 ^1^
	Contralateral	Hippocampus			
			Control	4 (3; 5)	
			Solvent control	3 (2; 5)	
			DHEA 0.1 µg/g bw	5 (4; 5)	
			DHEA 1 µg/g bw	5 (3; 6)	
			DHEA 10 µg/g bw	4 (2; 6)	
			DHEAS 0.1 µg/g bw	5 (3; 9)	
			DHEAS 1 µg/g bw	4 (4; 6)	
			DHEAS 10 µg/g bw	4 (3; 6)	0.882 ^1^
	Ipsilateral	Hippocampus			
			Control	69 (23; 87)	
			Solvent control	35 (5; 96)	
			DHEA 0.1 µg/g bw	48 (25; 109)	
			DHEA 1 µg/g bw	81 (4; 107)	
			DHEA 10 µg/g bw	41 (8; 115)	
			DHEAS 0.1 µg/g bw	53 (8; 159)	
			DHEAS 1 µg/g bw	51 (7; 112)	
			DHEAS 10 µg/g bw	70 (49; 135)	0.951 ^1^
	Contralateral	Thalamus			
			Control	6 (5; 15)	
			Solvent control	6 (4; 11)	
			DHEA 0.1 µg/g bw	7 (4; 12)	
			DHEA 1 µg/g bw	6 (5; 10)	
			DHEA 10 µg/g bw	15 (6; 25)	
			DHEAS 0.1 µg/g bw	9 (6; 12)	
			DHEAS 1 µg/g bw	7 (4; 8)	
			DHEAS 10 µg/g bw	7 (4; 16)	0.680 ^1^
	Ipsilateral	Thalamus			
			Control	43 (15; 113)	
			Solvent control	16 (6; 64)	
			DHEA 0.1 µg/g bw	12 (6; 29)	
			DHEA 1 µg/g bw	35 (8; 65)	
			DHEA 10 µg/g bw	10 (6; 41)	
			DHEAS 0.1 µg/g bw	15 (4; 38)	
			DHEAS 1 µg/g bw	8 (5; 22)	
			DHEAS 10 µg/g bw	23 (10; 39)	0.381 ^1^
Frontal	Contralateral	White matter			
			Control	6 (4; 10)	
			Solvent control	6 (5; 10)	
			DHEA 0.1 µg/g bw	8 (6; 9)	
			DHEA 1 µg/g bw	11 (7; 14)	
			DHEA 10 µg/g bw	4 (4; 8)	
			DHEAS 0.1 µg/g bw	5 (3; 10)	
			DHEAS 1 µg/g bw	5 (4; 18)	
			DHEAS 10 µg/g bw	8 (5; 13)	0.494 ^1^
	Ipsilateral	White matter			
			Control	22 (9; 52)	
			Solvent control	9 (4; 30)	
			DHEA 0.1 µg/g bw	8 (7; 27)	
			DHEA 1 µg/g bw	35 (12; 62)	
			DHEA 10 µg/g bw	6 (4; 17)	
			DHEAS 0.1 µg/g bw	16 (3; 67)	
			DHEAS 1 µg/g bw	7 (2; 30)	
			DHEAS 10 µg/g bw	14 (6; 29)	0.409 ^1^
	Contralateral	Cortex			
			Control	16 (13; 30)	
			Solvent control	17 (9; 20)	
			DHEA 0.1 µg/g bw	23 (17; 32)	
			DHEA 1 µg/g bw	16 (10; 22)	
			DHEA 10 µg/g bw	15 (10; 17)	
			DHEAS 0.1 µg/g bw	15 (11; 22)	
			DHEAS 1 µg/g bw	16 (14; 18)	
			DHEAS 10 µg/g bw	14 (9; 24)	0.468 ^1^
	Ipsilateral	Cortex			
			Control	89 (24; 231)	
			Solvent control	26 (9; 107)	
			DHEA 0.1 µg/g bw	23 (16; 93)	
			DHEA 1 µg/g bw	145 (24; 204)	
			DHEA 10 µg/g bw	13 (9; 68)	
			DHEAS 0.1 µg/g bw	54 (9; 184)	
			DHEAS 1 µg/g bw	21 (15; 85)	
			DHEAS 10 µg/g bw	59 (24; 92)	0.351 ^1^
	Contralateral	Striatum			
			Control	6 (4; 10)	
			Solvent control	6 (3; 8)	
			DHEA 0.1 µg/g bw	9 (7; 13)	
			DHEA 1 µg/g bw	9 (4; 11)	
			DHEA 10 µg/g bw	8 (6; 11)	
			DHEAS 0.1 µg/g bw	6 (4; 9)	
			DHEAS 1 µg/g bw	5 (4; 9)	
			DHEAS 10 µg/g bw	5 (4; 6)	0.305 ^1^
	Ipsilateral	Striatum			
			Control	92 (8; 198)	
			Solvent control	13 (5; 104)	
			DHEA 0.1 µg/g bw	12 (9; 117)	
			DHEA 1 µg/g bw	130 (15; 288)	
			DHEA 10 µg/g bw	10 (4; 15)	
			DHEAS 0.1 µg/g bw	15 (4; 72)	
			DHEAS 1 µg/g bw	9 (5; 85)	
			DHEAS 10 µg/g bw	16 (10; 69)	0.296 ^1^

The occipital section plane corresponds to coronal level 72 (bregma −1.755 mm), frontal section plane corresponds to coronal level 44 (bregma 1.045 mm). *Abbreviations: DHEA*, dehydroepiandrosterone; *DHEAS*, dehydroepiandrosterone sulfate; *bw*, body weight. ^1^ Overall statistical differences were assessed using the Kruskal–Wallis test.

## Data Availability

The original contributions presented in the study are included in the article; further inquiries can be directed to the corresponding author.

## Supplementary Materials

The following supporting information can be downloaded at: https://www.mdpi.com/article/10.3390/antiox13121542/s1, Table S1: Materials and substances used in the study.
