# Supplementary material for: Effects of DHEA and DHEAS in Neonatal Hypoxic–Ischemic Brain Injury
Source: Antioxidants (Basel). 2024 Dec 16;13(12):1542. doi: 10.3390/antiox13121542 (PMC11726961; doi:10.3390/antiox13121542)
Supplement: Supplementary file 1 [file antioxidants-13-01542-s001.zip › antioxidants-3305661-supplementary.pdf]

Supplementary Table S1: Materials and substances used in the study.

**Material/Substance**	**Catalogue Number**	**Company**
Animal experiments		
Emla® 5% (lidocaine/prilocaine)	PZN 13231250	Aspen Pharma Ireland Ltd, Ireland
Forene® Isoflurane		Abbott GmbH, Germany
DHEA	709549	Sigma Aldrich, Germany
DHEAS	723266	Sigma Aldrich, Germany
Immunohistochemical analyses		
Anti cleaved caspase 3 antibody	9664	Cell Signaling Technology, USA
Anti IBA-1 antibody	013-27691	Wako, China
Goat anti rabbit secondary antibody	JAC111065003	Jackson Immunoresearch; Szabo Scandic, Austria
VECTASTAIN Elite ABC Kit	PK-6100	VectorLabs; Szabo Scandic, Austria
DAB substrate Kit	34002	Pierce/Fisher Scientific GmbH, Austria
Protein fractionation and Western Blot		
1x complete EDTA free protease inhibitor cocktail	04693132001	Roche, Austria
PMSF	93482	Sigma Aldrich, Germany
BCA Protein Assay Kit	23227	Pierce/Fisher Scientific GmbH, Austria
4-20% Mini Protean® TGX Stain-Free™ Protein Gels	456-8095	Biorad, Germany
Immobilon-PSQ PVDF Membrane	ISEQ00010	Merck Millipore, France
Anti SOD1 antibody	67480-1-Ig	Proteintech/THP, Austria
Anti NOX2 antibody	19013-1-AP	Proteintech/THP, Austria
FastGene Western ECL Kit	FG-CH01	Nippon Genetics Europe, Germany
Anti β-Actin, clone AC-74	A5316	Sigma Aldrich, Germany
ELISA		
4-HNE ELISA	ELSE-EL-0128-96	Elabscience/Szabo Scandic, Austria
8-OHdG ELISA	STRSKT-120-96S	StressMarq/Szabo Scandic, Austria
